# A Commentary on: “Preserving cortico-striatal function: Deep Brain Stimulation in Huntington's disease”

**DOI:** 10.3389/fnsys.2015.00057

**Published:** 2015-04-14

**Authors:** Christopher M. Tolleson

**Affiliations:** Department of Neurology, Vanderbilt UniversityNashville, TN, USA

**Keywords:** Huntington's disease, Deep Brain Stimulation, electrostimulation, neuroprotection, globus pallidus

I read Nagel et al. with interest (Nagel et al., 2015). In theory, the potential to modify cognitive function in Huntington's disease (HD) with electrical stimulation or specifically Deep Brain Stimulation (DBS) is exciting. HD is a devastating and terminal disease in need of better treatments, both symptomatic and neuroprotective. Cognitive dysfunction itself is becoming more recognized as a severely disabling aspect of the disease as well (Paulsen and Long, 2014). Nagel et al. outline an intriguing argument for pursuing the globus pallidus externa as a DBS target for cognitive improvement while also acknowledging that the current evidence is based on a small number of animal models and very limited human (*n* = 2) experience.

In actuality, the practical nature of pursing DBS for the treatment of cognition in HD is more complicated. The ethics of pursuing DBS for new indications is a hotly debated topic (Unterrainer and Oduncu, 2015). We still do not yet fully understand the mechanism of DBS in treating disorders where its efficacy is established. Trialing it for other neurologic conditions without sound rational can be unnecessarily dangerous and not in the best interest of those we are trying to treat. While DBS was relatively free of complications in limited trials for HD chorea, it is not a benign procedure. It is a brain surgery with known irreversible surgical complications including infection, stroke, intracranial bleeding and even death (Bronstein et al., 2011). Some authors cite complication rates in Parkinson's disease as high as 10% for hemorrhage, 2% for stroke, 15% for infection and death rates up to 4.5% (Bronstein et al., 2011). Hardware complications requiring repeat surgery also occur. Finally, cognition and behavior have also been known to be negatively impacted after DBS surgery, albeit more often cited in the subthalamic target for Parkinson's disease. Still, there are accounts of cognitive decline and even mania after pallidal DBS as well (Miyawaki et al., 2000; Rothlind et al., 2014). Considering these known potential neuropsychiatric complications, trials of DBS for HD would need to proceed very cautiously given the high incidence of pre-existing cognitive dysfunction and increased risk for suicide.

Lastly, I would caution against making any statements alluding to slowing disease progression or neuroprotection given the current data, especially to perspective future participants in any DBS trials. This is too premature. This is indeed a desperate population and the ability to identify subjects early in the prodromal stage of the disease increases the potential for inappropriate and improper recruitment to any future trials. Any research using DBS in this population needs to be done slowly with the highest ethical standards and rigorous consenting protocols. Personally, while I commonly recommend DBS for my patients for treatment of currently approved indications and participate in a high volume of surgeries, I am still holding out hope for a medicinal, neuroprotective therapy that would have reversible adverse effects and not carry the risk of a brain surgery.

## Conflict of interest statement

The author declares that the research was conducted in the absence of any commercial or financial relationships that could be construed as a potential conflict of interest.
